# Viability and Stimulation of Human Stem Cells from the Apical Papilla (hSCAPs) Induced by Silicate-Based Materials for Their Potential Use in Regenerative Endodontics: A Systematic Review

**DOI:** 10.3390/ma13040974

**Published:** 2020-02-21

**Authors:** José Luis Sanz, Leopoldo Forner, Alicia Almudéver, Julia Guerrero-Gironés, Carmen Llena

**Affiliations:** 1 Dental Pathology and Therapeutics Unit, Department of Stomatology, Universitat de València, 46010 Valencia, Spain; jsanzalex96@gmail.com (J.L.S.); aag1094@gmail.com (A.A.); llena@uv.es (C.L.); 2 Special Care and Gerodontology Unit, Department of Stomatology, University of Murcia, 30100 Murcia, Spain; juliaguerrero1@hotmail.com

**Keywords:** silicate-based materials, human stem cells from the apical papilla, regenerative endodontic treatment

## Abstract

Blood clot formation in the apical third of the root canal system has been shown to promote further root development and reinforcement of dentinal walls by the deposition of mineralized tissue, resulting in an advancement from traditional apexification procedures to a regenerative endodontic treatment (RET) for non-vital immature permanent teeth. Silicate-based hydraulic biomaterials, categorized as bioactive endodontic cements, emerged as bright candidates for their use in RET as coronal barriers, sealing the previously induced blood clot scaffold. Human stem cells from the apical papilla (hSCAPs) surviving the infection may induce or at least be partially responsible for the regeneration or repair shown in RET. The aim of this study is to present a qualitative synthesis of available literature consisting of in vitro assays which analyzed the viability and stimulation of hSCAPs induced by silicate-based hydraulic biomaterials. A systematic electronic search was carried out in Medline, Scopus, Embase, Web of Science, Cochrane and SciELO databases, followed by a study selection, data extraction, and quality assessment following the PRISMA protocol. In vitro studies assessing the viability, proliferation, and/or differentiation of hSCAPs as well as their mineralization potential and/or osteogenic, odontogenic, cementogenic and/or angiogenic marker expression in contact with commercially available silicate-based materials were included in the present review. The search identified 73 preliminary references, of which 10 resulted to be eligible for qualitative synthesis. The modal materials studied were ProRoot MTA and Biodentine. Both bioceramic materials showed significant positive results when compared to a control for hSCAP cell viability, migration, and proliferation assays; a significant up-regulation of hSCAP odontogenic/osteogenic marker (ALP, DSPP, BSP, Runx2, OCN, OSX), angiogenic growth factor (VEGFA, FIGF) and pro-inflammatory cytokine (IL-1α, IL-1β, IL-6, TNF-α) expression; and a significant increase in hSCAP mineralized nodule formation assessed by Alizarin Red staining. Commercially available silicate-based materials considered in the present review can potentially induce mineralization and odontogenic/osteogenic differentiation of hSCAPs, thus prompting their use in regenerative endodontic procedures.

## 1. Introduction

The introduction of regenerative endodontic treatment (RET) as an alternative approach to the established apexification procedures for the treatment of non-vital immature permanent teeth has resulted in an important development of the current paradigm in endodontic therapy [1]. The so-called “revascularization” produced in RET results from the orthograde extrusion of an endodontic file beyond the apical foramen, and the subsequent induction of bleeding from the periapical tissue [2]. Blood clot formation in the apical third has been shown to promote further root development and reinforcement of dentinal walls by the deposition of mineralized tissue [3].

The characterization of stem cells from the apical papilla (SCAPs) gave rise to a plausible explanation for this phenomenon. SCAPs were categorized as multipotent mesenchymal stem cells (MSCs) after a positive expression of STRO-1, CD146, CD73, CD90, and CD105 markers. [4,5,6]. It has been suggested that MSCs may be present virtually in any vascularized tissue [7], so the expression of the aforementioned markers, indicative of MSC nature, reflect the perivascular location and multilineage differentiation potential of SCAPs [8]. In addition, it has been described that SCAPs are capable of exhibiting a variety of osteo/dentinogenic markers (BSP, DSPP, ALP, Runx2, MEPE) [9,10,11], and reports have shown different degrees of root maturation after disinfection in cases with severely infected pulps in immature teeth [12,13,14]. Altogether, this led to suggest the possibility that remaining SCAPs in the apical papilla surviving the infection may induce or at least be partially responsible for the mineralized tissue formation or repair shown in RET [15,16].

Reported RET protocols share three main components: disinfection of the root canals, recruitment of MSCs and scaffold establishment, and placement of a coronal barrier and restoration [17]. However, they vary in terms of invasiveness (i.e., degree and extent of instrumentation of the root canals), disinfection or irrigation sequence, biomaterial used as a scaffold and coronal barrier (if any), and final restoration placed [18].

Regarding coronal barriers, properties like biocompatibility or absence of cytotoxicity are critical for their use in RET, since they will be in direct contact with the apical blood clot serving as a scaffold [19]. Ideally, biomaterials used for this purpose should also express antibacterial and bioactive properties, in order to ensure the survival and promote differentiation of the remaining MSCs after infection and disinfection [20]. The term bioactivity commonly refers to the liberation of OH^−^ and Ca^2+^ ions which interact with the mineralized constituent of dentinal tissue in order to form a mineral attachment to the dentin substrate [21].

Silicate-based hydraulic biomaterials, categorized as bioactive endodontic cements (BECs), emerged as bright candidates for the coronal sealing of the previously established blood clot scaffold [22]. These non-resorbable biocompatible materials have shown bioactive properties in direct contact with dental pulp stem cells (DPSCs) using in vitro assays [23]. Mineralization ability or bioactivity of bioceramic materials is most commonly measured by using quantitative reverse-transcriptase polymerase chain reaction (qRT-PCR) to quantify the expression of osteogenic, odontogenic, and cementogenic markers; and alizarin red staining (ARS) to analyze the extent of mineralized deposits produced [24,25].

Currently, available literature tends to assess the differences between recently introduced silicate-based materials like Biodentine (BD; Septodont, Saint Maurdes-Fosses, France) with the established mineral trioxide aggregate (MTA) or even traditional coronal barriers like calcium hydroxide (CH) [26,27,28].

Taking into account the desirable properties expressed by silicate-based materials in contact with dental pulp stem cells and the reported multipotentiality and convenient location of stem cells from the apical papilla (SCAPs), it seems pertinent to provide an updated vision of the interrelation between them for their potential use in regenerative endodontic procedures.

Within this framework, this study aims to present a qualitative synthesis or systematic review of available literature consisting of in vitro assays which analyzed the viability and stimulation of human stem cells from the apical papilla (hSCAPs) induced by silicate-based hydraulic biomaterials or bioceramic materials.

## 2. Materials and Methods

Data from the present work were presented in accordance with the PRISMA guidelines or preferred reporting items for systematic reviews and meta-analysis [29].

Our research question, based on the PICOS model [30], aimed to explore the potential use of silicate-based hydraulic biomaterials as coronal barriers in regenerative endodontic procedures for the treatment of non-vital immature permanent teeth, by synthesizing the methodology and results of studies assessing the viability and stimulation of SCAPs when placed in direct contact with the said materials using in vitro assays.

### 2.1. Inclusion and Exclusion Criteria

In vitro studies assessing the viability, proliferation, and/or differentiation of SCAPs or cells from the apical papilla (APCs) as well as their mineralization potential and/or osteogenic, odontogenic, cementogenic, and/or angiogenic marker expression in contact with commercially available silicate-based materials were included in the present review. Both assays comparing the variables mentioned above between one or more silicate-based materials or between a silicate-based material and a control group were accepted. Studies assessing only one silicate-based material were also included. The comparison of the previously described variables between different types of DSCs or stem cells other than SCAPs was considered as a reason for exclusion.

Criteria for inclusion and exclusion were settled by a consensus reached from all authors, considering the research question and objectives of the study, while attempting to obtain an ample range of results from the search strategy.

### 2.2. Search Strategy

#### 2.2.1. Sources of Information

In order to establish potentially eligible studies, a systematic electronic search was carried out in Medline, Scopus, Embase, Web of Science, Cochrane, and SciELO databases. The search was conducted during October 2019 and updated in December 2019. Both the search and data extraction were carried out by two independent examiners, and in case of any discrepancy, a third examiner was consulted.

#### 2.2.2. Search Terms

**Figure 1:**
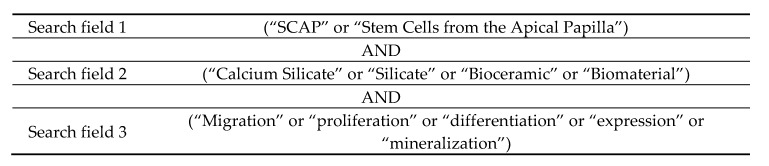
Search strategy illustration.

The search strategy included three Mesh (Medical Subject Heading) terms: “silicate,” “calcium silicate,” or “biomaterial” and eight uncontrolled descriptors: “SCAP,” “stem cells from the apical papilla,” “bioceramic,” “migration,” “proliferation,” “differentiation,” “expression,” and “mineralization”. Boolean operators (“OR” and “AND”) were used to annex the search terms related to the search question (Figure 1).

#### 2.2.3. Study Selection

References identified using the previously mentioned search terms were exported to Mendeley reference manager software (Elsevier, Amsterdam, Netherlands) to check for duplicates. After discarding duplicates, record titles and abstracts were screened according to the inclusion and exclusion criteria. Studies that met the criteria where then assessed for eligibility for qualitative synthesis by full-text screening.

#### 2.2.4. Study Data

Data synthesis resulted from an extraction of a series of variables for methodology and results from the included studies. Variables extracted for methodology were: the stem cell variant used and its origin or source, the bioceramic materiall used and its/their concentration, and the activity analysis carried out and its duration. Variables recorded for results were: the significant results found, the time at which they were recorded (duration), and their significance level. In the case of assays analyzing the expression of different markers, results were divided and presented for each marker.

### 2.3. Quality Assessment

Risk of bias of the included studies was analyzed using a modified CONSORT checklist of items for reporting in vitro studies of dental materials [31], assessing the fulfillment for each of the quality assessment parameters or items considered in the checklist.

## 3. Results

### 3.1. Study Selection and Flow Diagram

**Figure 2:**
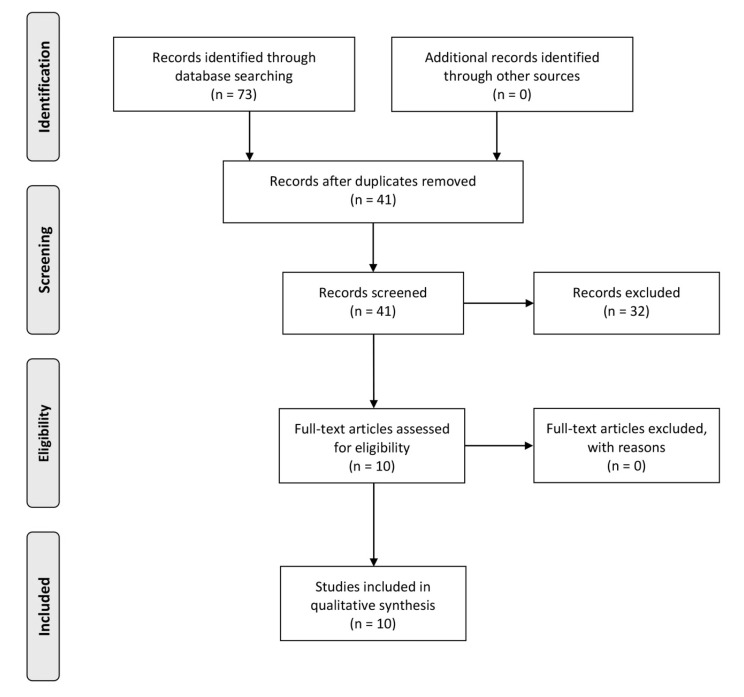
Systematic flow-chart representing study inclusion.

The search identified 73 preliminary references related to the influence of bioceramic materials on hSCAPs, of which 38 were found in Medline, 7 in Scopus, 15 in Embase, 11 in Web of Science, and 1 in SciELO. The search carried out in the Cochrane database produced no results. After discarding 32 duplicates, the resulting 41 records were screened. Of these, 31 were excluded from reading the title and abstract, as they did not fulfill the inclusion criteria. The remaining ten articles were assessed at a full-text level. All ten articles resulted in being eligible for qualitative synthesis (Figure 2).

### 3.2. Study Characteristics

#### 3.2.1. Cell Variant and Origin

A summary of the methodology used by the studies included in the review is shown in Table 1.

Eight out of the ten included studies used human stem cells from the apical papilla (hSCAPs) from impacted immature third molars as their cell variant for analysis [32,33,34,35,36,38,39,41]. One of the studies used hSCAPs as their cell variant but did not specify its dental origin [40]. The remaining study used cells from the apical papilla (APCs) [37]. Cell variants and their origin are presented in Table 1.

#### 3.2.2. Bioceramic Materials Used and Concentration

Commercially available silicate-based materials assessed in the included studies are presented in Table 2. The concentrations used for said bioceramic materials are presented in Table 1.

#### 3.2.3. Activity Analysis

Analyses carried out in the study sample were subdivided into three categories according to the outcome measured. The first category corresponds to the analyses measuring hSCAP cell viability, migration, and proliferation, for which a wide range of assays were used: flow cytometry [33,41], MTT assay [32,36], wound healing assay [32,35], transwell migration assay [32,40]; cell proliferation assays using trypan blue [33], WST-1 [40], Coulter counter [41], and BrdU labeling [32]; and cell viability assays using OZBlue [34], Alamar blue [37], CCK-8 [38], and XTT [39]. The second category was reserved for those analyses quantifying hSCAP expression of activity-related markers, majorly carried out using qRT-PCR [32,33,34,35,36,38,39,41], and followed by Western blot [32,38] and ELISA [38]. Analyses of the mineralization potential of hSCAPs represented the third category and were carried out exclusively using alizarin red staining (ARS) [32,33,34,35,36,38,41]. Activity analyses alongside with their duration and a description of the study associated with them are presented in Table 1.

### 3.3. Quality Assessment

All in vitro studies assessed using the modified CONSORT checklist [31] (Table 3) reported an organized abstract (item 1) and an introduction which presented adequate background information about the silicate-based material/s and activity assays studied (2a), but only three of the ten studies presented explicit objectives and hypotheses (item 2b). Methodology description and variable synthesis were enough to allow for replication in all of the studies (items 3 and 4). However, the calculation of the sample size and a mention of the allocation sequence used (if any) was obviated in all of the studies (items 5–9). The statistical method used was reported in all of the studies (item 10), but only one of them presented significance levels as confidence intervals and not p values (item 11). Regarding the items referring to the discussion, studies tended to include a brief report of the critical results and compare them with related findings from other published papers, but only three of the ten studies mentioned their possible limitations (item 12). All studies noted their sources of funding (if any) (item 13), and no references to full trial protocols were included in any of the studies (item 14).

### 3.4. Study Results

#### 3.4.1. Results for hSCAP Cell Viability, Migration, and Proliferation Assays

Results for cell viability, migration, and proliferation assays (Table 4) comparing a bioceramic material with mineral trioxide aggregate (PR MTA/R MTA) showed mixed results (iRoot FS > PR MTA using a transwell migration assay; PR MTA > iRoot FS using a wound healing assay [32]).

The comparison of bioceramic materials with a control produced both positive significant results (PR MTA: using a transwell migration assay [32], Trypan Blue cell proliferation assay [33], MTT assay [36], XTT cell viability assay [39], and WST cell proliferation assay [40]; BD: using Trypan Blue cell proliferation assay [33], OZ Blue cell viability assay [34], MTT assay [36] and XTT cell viability assay [39]; iRoot FS: using a transwell migration assay [32]; R MTA: using MTT assay [36]; OCP: using flow cytometry [33]; ES: using OX Blue cell viability assay [34]; Atlantik: using Trypan Blue cell proliferation assay [33]) and negative significant results (PR MTA: using a wound healing assay [32], and OZ Blue cell viability assay [37]; BD: using Alamar Blue cell viability assay and a wound-healing assay [37]; iRoot FS: using a wound-healing assay [32]; OCP, CEM: using trypan blue cell proliferation assay [33]), depending on the silicate-based material studied.

#### 3.4.2. Results for the Quantification of hSCAP Activity-Related Marker Expression

Results for activity-related marker expression using RT-PCR (Table 5) comparing a silicate-based material with mineral trioxide aggregate (PR MTA/R MTA) showed significant positive results iRoot FS [32], and mixed results depending on the marker being assessed (CEM, Atlantik, OCP [33]; ES, ES FS [34]; BD [33,36,39]).

The comparison of silicate-based materials with a control resulted in majorly positive significant results for the bioceramic materials (PR MTA [32,33,34,41]; BD [33,34]; iRoot FS [32]; iRoot FM [38]; CEM [33]; ES [34]), or mixed results depending on the studied marker (PR MTA [35,39]; OCP, Atlantik [33]; CEMb [35]; BD [39]).

Protein expression using Western blot revealed significant mineralization results for iRoot FM compared to a control [38], and ELISA produced mixed results for PR MTA and BD compared to a control [39].

#### 3.4.3. Results for hSCAP Mineralization Potential Assays

Results for alizarin red staining or ARS (Table 6) comparing a silicate-based material with mineral trioxide aggregate (PR MTA/R) showed MTA positive significant results for the studied bioceramic materials (iRoot FS [32]; BD [36]).

The comparison of bioceramic materials with a control resulted in exclusively positive significant results for the silicate-based materials (PR MTA [32,33,34,39]; BD [33,34]; iRoot FS [32]; iRoot FM [38]; CEM, Atlantik, OCP [33]; ES [34]).

## 4. Discussion

Following the aim of the present review, a qualitative synthesis or systematic review of available literature analyzing the viability and stimulation of hSCAPs induced by commercially available silicate-based hydraulic biomaterials was presented.

This systematic review was not eligible for registration in the PROSPERO database for the international prospective international register of systematic reviews, as it currently does not consider systematic reviews based on in vitro studies.

A total of eleven different commercially available silicate-based materials were considered in the review (as shown in Table 2). The modal materials studied were ProRoot MTA (PR MTA), addressed in nine studies [32,33,34,35,36,37,39,40,41], and Biodentine (BD), approached in five studies [33,34,36,37,39]. It may be worth noting that studies assessing BD also included PR MTA in their sample, thereby allowing to consider PR MTA as the reference material for comparison.

Regarding PR MTA, significant results for hSCAP cell viability, migration, and proliferation assays showed both positive [32,33,36,39,40] and negative [32,37] outcomes for the bioceramic material when compared to a control. The same occurred with BD, showing both positive [33,34,36,39] and negative [37] outcomes. Whether the positive results outweigh the negative ones is unclear, considering that different types of methodologically dissimilar assays were carried out by each of the studies.

With reference to hSCAP activity-related marker expression, PR MTA has shown an up-regulation of a series of odontogenic/osteogenic genes (ALP [32,33,35,41]; DSPP [32,33]; Runx2, OCN [33,41]; OSX/SP7 [33,35]; BSP [33,34]) when compared to a control. BD reported a similar pattern, increasing the expression of various odontogenic/osteogenic markers (BSP [33,34]; OCN, OSX, Runx2, ALP, DSPP [33]). The up-regulation of these markers denotes their capability to induce odontogenic/osteogenic differentiation of hSCAPs.

However, angiogenic growth factor release showed mixed results for the studied bioceramic materials. Both PR MTA and BD reported a significant up-regulation of VEGFA and FIGF while down-regulating the expression ANGPT1 and FGF2 [39], suggesting a possible partial mediation in angiogenesis.

A significant up-regulation of pro-inflammatory cytokine release from hSCAPs has also been seen for both PR MTA (IL-1α, IL-1β, IL-6 [33,41]; TNF-α [33]) and BD (IL-1α, IL-1β, IL-6, TNF-α [33]). Pro-inflammatory cytokine release has been associated with the activation of the NF_K_B pathway, involved in the regulatory induction of odontogenesis/osteogenesis by DSP, Runx2, BMP2 and OSX [33]; and therefore, the upregulation shown by PR MTA and BD illustrates their potential to favor this differentiation.

When assessing hSCAP mineralization potential in contact with silicate-based materials, alizarin red staining was used by all of the studies to evaluate the formation of calcium deposits or mineralized nodules. All studies assessing PR MTA produced significant positive results for the bioceramic material when compared to a control group [32,33,34,39]; except in one case, in which the difference was not significant [36]. BD, however, produced exclusively positive significant results compared to a control group in all of the ARS assays carried out [33,34,36]. The reported increase in calcium nodule formation implies that both of these bioceramic materials can potentially induce mineralization in direct contact with hSCAPs.

Comparisons between the previously mentioned materials were also carried out [33,34,36,37,39]. Both bioceramic materials performed similarly in hSCAP cell viability, migration, and proliferation assays, favoring PR MTA in two cases (transwell migration assay, Alamar blue cell viability assay [37]). For hSCAP activity-related marker expression, BD reported a significant up-regulation of a series of osteogenic/odontogenic (DSPP [33,36]; ALP [34]; BSP [33], DMP-1, MEPE [36]) and angiogenic (TGFβ1 [39]) markers when compared to PR MTA, while producing a significantly lower up-regulation of other osteogenic/odontogenic markers (OCN [33,36]; OSX, DSPP [33]; BSP [34]) and angiogenic growth factors (ANGPT-1, FGF2 [39]). Reported mineralized nodule formation using ARS was similar for both materials, favoring BD, in one case [36]. In this context, reaching conclusions about whether one material is superior to the other would be noticeably inconsequential, considering the heterogeneity of both the methodology used by the included studies and the results reported.

The remaining nine silicate-based materials contemplated in this review (iRoot FS, iRoot FM, R MTA, CEM cement, ES, ES FS, OCP, Atlantik, PG) were only studied once. Consequently, aside from the descriptive qualitative synthesis presented previously, limited conclusions can be drawn: iRoot FS, OCP, Atlantik, ES, and iRoot FM reported positive significant results for at least one hSCAP cell viability, proliferation or migration assay; a significant up-regulation of hSCAP expression of at least one osteogenic/dentinogenic marker; and a significant increase in mineralized nodule formation using ARS in comparison with a control group.

The nature of the control groups used for comparison was specified by all of the included studies, distinguishing between negative and positive control groups. Generally, results from the different biocompatibility and activity assays were presented using a negative control group as a reference [32,33,34,36,37,38,39,40,41]. hSCAPs cultured in culture media acted as negative control groups, varying between the studies. Alpha minimum essential medium (α-MEM) was used by the majority of the studies [32,36,38,39,40,41], either plain [40] or with supplements [32,36,38,39,41]. Other media used were Dulbecco’s modified Eagle medium (DMEM) [33], apical papilla cell (APC) culture media [37], and dentin disks [34]. Supplements used included fetal bovine serum (FBS) at different concentrations [32,33,35,36,37,38,41], penicillin and streptomycin [32,33,37,38,39,41], and L-glutamine [36,39]. A positive control group was used as a comparison in one case [35], consisting of hSCAPs cultured in a high glucose DMEM supplemented with osteogenic reagents. Differences in culture media characteristics hinder the interpretation and comparison of the results produced, highlighting the need for the use of standardized procedures in future studies.

Regarding bioceramic material concentrations, included studies followed various routes. Material dosage was established using ALP enzyme activity assays in two cases [33,41], categorizing the ratio or concentration, which produced the highest concentration of ALP as optimal and using it for further activity assays. In a similar manner, a CCK-8 assay for the assessment of cell proliferation was used in one case for the same purpose [38]. One study selected the optimal concentration from previous evidence [32], and the remaining studies reported a biomaterial preparation following the manufacturer’s instructions [34,35,37,39,40].

Various concentrations were assessed for PR MTA and BD. Those which produced positive significant results when compared to a control for both hSCAP cell viability, migration, proliferation, activity, and mineralization assays were: 2mg/mL [32] and 0.2mg/mL [33] for PR MTA, and 2mg/mL [33] and 0.15mg/mL [36] for BD. This range of potentially optimal concentrations may be useful as a reference for future studies, since the influence of bioceramic materials on hSCAP activity assays has been described as dose-dependent [33,38,41]. However, the individual establishment of the highest activity-inducing concentration as optimal using activity assays e.g., ALP activity assay and/or cell proliferation assays e.g., CCK-8 assay, is ideal.

To the authors’ knowledge, this is the first systematic review assessing the influence of silicate-based materials on human stem cells from the apical papilla. Considering the scarcity and in vitro characteristics of the available evidence in this matter, extrapolation of the results obtained to a clinical level is far from applicable. However, having illustrated the positive influence of the studied bioceramic materials on these cells, it would be convenient to advance into in vivo trials and broaden the spectrum of assays performed on different conditions without sacrificing uniformity in the methodology used, to allow for a posterior collective analysis of the evidence.

## 5. Conclusions

Commercially available silicate-based materials considered in the present review can potentially induce mineralization and odontogenic/osteogenic differentiation of hSCAPs, thus prompting their use in regenerative endodontic procedures.

## Figures and Tables

**Table 1:** Summary of the methodology of included studies.

Author	Cell Type and Origin	Bioceramics Used (concentration *)	Activity Analysis **	Duration
Liu et al. 2019 [32]	hSCAP from impacted immature third molars	iRoot FS (2 mg/mL), PR MTA (2 mg/mL)	Wound healing assay (DSPP, ALP)	12, 24 h
			BrdU labeling assay	20 h
			MTT assay	1, 2, 3, 4 days
			Transwell migration assay	24 h
			qRT-PCR (DSPP, ALP)	6 days
			Western blot analysis	6 days
			Alizarin red staining	4 weeks
Saberi et al. 2019 [33]	hSCAP from impacted immature third molars	PR MTA (200 μg/mL), BD (2 mg/mL), CEM (20 mg/mL), Atlantik (20 μg/mL), OCP (200 μg/mL)	Trypan blue cell proliferation assay	1, 3, 5, 7, 9 days
			Flow cytometry	5 days
			ALP activity	72 h
			Alizarin red staining	21 days
			qRT-PCR (ALP, DSPP, RUNX2, OSX, OCN, BSP, TNF-α, IL-Iα, IL-Iβ, IL-6)	3, 7 days
Miller et al. 2018 [34]	hSCAP from mandibular immature third molar	PR MTA, BD, ES, ES-FS	OZBlue cell viability assay	7 days
			qRT-PCR (ALP, DSPP, IBSP, Runx2)	21 days
			Alizarin red staining	21 days
Hajizadeh et al. 2018 [35]	hSCAP from impacted immature third molar	PR MTA, CEM	Alizarin red staining	2, 3 weeks
			qRT-PCR (ALP, DSPP, OSC, SP7)	2, 3 weeks
Wongwatanasanti et al. 2018 [36]	hSCAP from mandibularimmature third molar	PR MTA(0.13 mg/mL), BD (0.14 mg/mL), R MTA (0.1 mg/mL)	MTT assay	1, 3, 7, 14 days
			Alizarin red staining	7, 14, 21 days
			qRT-PCR (OCN, DSPP, MEPE, DMP-1)	1, 7, 14, 21 days
Sequeira et al. 2018 [37]	APC from immature third molars	PR MTA, BD, PG	Alamar blue cell viability assay	21, 48, 72 h
			Wound healing assay	0, 24, 28 h
Bi et al. 2018 [38]	hSCAP from impacted immature third molars	iRoot FM (0.5 mg/mL)	CCK-8 cell viability assay kit	1, 3, 5 days
			qRT-PCR (DMP-1, ALP)	10 days
			Western blot analysis (DMP-1, ALP)	10 days
			Alizarin red staining	4 weeks
Peters et al. 2015 [39]	hSCAP from immature third molars	PR MTA, BD	XTT cell viability assay kit	1, 3, 7 days
			ELISA (VEGF, ANGPT1)	1, 3 days
			qRT-PCR (VEGFA, VEGFC, FIGF, ANGPT1, ANG, FGF2, TGFB1, MMP2, IL8, TIMP2)	3 days
Schneider et al. 2015 [40]	hSCAP	PR MTA (100 mg/35 μL)	Transwell migration assay	1, 3, 6, 12, 24, 48, 72 h
			WST-1 proliferation assay	1, 3, 5, 7, 9, 11, 14 days
Yan et al. 2014 [41]	hSCAP from immature third molars	PR MTA (2 mg/mL)	Coulter counter cell proliferation assay	1, 3, 5, 7, 9 days
			Flow cytometry	5 days
			ALP activity	3 days
			Alizarin red staining	14 days
			qRT-PCR (ALP, DSPP, RUNX2, OSX, OCN, BSP, TNFα, IL-1α, IL-1β, IL-6)	3, 7 days

* Concentration of the bioceramic materials used is expressed in micrograms (μg) or milligrams (mg) per milliliter (mL) or microliter (μL), if specified by the authors; ** Genes, markers and/or proteins studied appear inside the parentheses; N/S: not specified.

**Table 2:** List of commercially available silicate-based materials studied.

Material	Abbreviation	Manufacturer	Times Studied
ProRoot MTA	PR MTA	Dentsply Tulsa Dental, Tulsa, OK, USA	9
Biodentine	BD	Septodont, Saint Maurdes-Fosses, France	5
iRoot Fast Set root repair material	iRoot FS	Dentsply Tulsa Dental, Tulsa, OK, USA	1
iRoot FM	−	Innovative Bioceramix Inc., Vancouver, BC, Canada	1
RetroMTA	R MTA	BioMTA, Seoul, Korea	1
CEM cement	CEM	NSK, Tokyo, Japan	1
	CEMb	BioniqueDent, Tehran, Iran	1
Endosequence BC Root Repair Material-Putty	ES	Brasseler, Savannah, GA, USA	1
Endosequence BC Root Repair Material-Putty fast set	ES FS	Brasseler, Savannah, GA, USA	1
Atlantik	−	Chemin du Catupolan, Vaulx en Velin, France	1
Octacalcium phosphate	OCP	N/S	1
PulpGuard	PG	Coltène-Whaledent, Altstätten, Switzerland	1

N/S: not specified.

**Table 3:** Results of the assessment of in vitro studies by the use of the modified CONSORT checklist [31]. Cells marked with an asterisk “*” represent study fulfillment for the given quality assessment parameter. Blank cells represent non-fulfillment.

Studies	Modified CONSORT Checklist of Items for Reporting in Vitro Studies of Dental Materials														
	1	2a	2b	3	4	5	6	7	8	9	10	11	12	13	14
Liu et al. 2019 [32]	*	*	−	*	*	−	−	−	−	−	*	−	−	*	−
Saberi et al. 2019 [33]	*	*	−	*	*	−	−	−	−	−	*	−	−	*	−
Miller et al. 2018 [34]	*	*	−	*	*	−	−	−	−	−	*	−	*	*	−
Hajizadeh et al. 2018 [35]	*	*	−	*	*	−	−	−	−	−	*	*	*	*	−
Wongwatanasanti et al. 2018 [36]	*	*	−	*	*	−	−	−	−	−	*	−	−	*	−
Sequeira et al. 2018 [37]	*	*	*	*	*	−	−	−	−	−	*	−	*	*	−
Bi et al. 2018 [38]	*	*	−	*	*	−	−	−	−	−	*	−	−	*	−
Peters et al. 2015 [39]	*	*	*	*	*	−	−	−	−	−	*	−	−	*	−
Schneider et al. 2015 [40]	*	*	−	*	*	−	−	−	−	−	*	−	−	*	−
Yan et al. 2014 [41]	*	*	*	*	*	−	−	−	−	−	*	−	−	*	−

**Table 4:** Summary of the results of included studies showing significant differences between bioceramic materials or a bioceramic material and a control for hSCAP cell viability, proliferation, and/or migration assays.

Author	Assay	Significant Results	Duration	Significance Level
Liu et al. 2019 [32]	Wound-healing assay	PR MTA > iRoot FS	12 h	*p* < 0.05
			24 h	*p* < 0.01
		Control > iRoot FS	12, 24 h	*p* < 0.01
		Control > PR MTA	24 h	*p* < 0.05
	Transwell migration assay	iRoot FS > PR MTA	24 h	*p* < 0.01
		iRoot FS > control	24 h	*p* < 0.005
		PR MTA > control	24 h	*p* < 0.05
Saberi et al. 2019 [33]	Cell proliferation assay (trypan blue technique)	PR MTA, BD, Atlantik > control	1 day	*p* < 0.05
		Control > OCP, CEM	3 days	*p* < 0.05
	Flow cytometry	OCP > control	5 days	*p* < 0.05
Miller et al. 2018 [34]	Cell viability assay (OZblue)	ES, BD > control	7 days	*p* < 0.05
		Control > PR MTA	7 days	*p* < 0.05
		ES > ES FS	7 days	*p* < 0.05
Wongwatanasanti et al. 2018 [36]	MTT assay	BD, PR MTA, R MTA > control	3, 7, 14 days	*p* < 0.05
Sequeira et al. 2018 [37]	Cell viability assay (Alamar blue)	PR MTA, PG, control > BD	24 h	*p* < 0.01
	Wound-healing assay		48, 72 h	*p* < 0.001
		PR MTA, PG, control > BD	24, 48 h	*p* < 0.05
Bi et al. 2018 [38]	CCK8 cell viability assay	iRoot FM > Ca(OH)_2_ *	3 days	*p* < 0.01
			5 days	*p* < 0.01
		iRoot FM > TAP **	3, 5 days	*p* < 0.005
Peters et al. 2015 [39]	XTT cell viability assay kit	PR MTA, BD > control	1 day	*p* < 0.05
Schneider et al. 2015 [40]	Transwell migration assay	PR MTA > control	6 h	*p* < 0.05
	WST-proliferation assay	PR MTA > control	1, 5 day	*p* < 0.05

* Ca(OH)_2_: 0.5 mg/mL calcium hydroxide; ** TAP: 0.01 mg/mL triple antibiotic paste.

**Table 5:** Summary of the results of included studies showing significant differences between bioceramic materials or a bioceramic material and a control for hSCAP activity-related marker expression.

Author	Analysis	Significant Results	Marker/Cytokine	Duration	Significance Level
Liu et al. 2019 [32]	qRT-PCR	iRoot FS > PR MTA	DSPP, ALP	6 days	*p* < 0.01
		iRoot FS > control		6 days	*p* < 0.005
		PR MTA > control		6 days	*p* < 0.01
Saberi et al. 2019 [33]	qRT-PCR	CEM, Atlantik > BD, PR MTA, OCP > control	BSP	3 days	*p* < 0.05
		BD > PR MTA, Atlantik > CEM > OCP > control		7 days	*p* < 0.05
		OCP > PR MTA > BD > CEM, Atlantik > control	OCN	3 days	*p* < 0.05
		PR MTA > OCP > BD, Atlantik > CEM > control		7 days	*p* < 0.05
		PR MTA, OCP > CEM > Atlantik > BD > control	OSX	3 days	*p* < 0.05
		PR MTA > OCP, BD > Atlantik > CEM > control		7 days	*p* <0.05
		Atlantik, PR MTA, CEM > BD > OCP, control	Runx2	3 days	*p* < 0.05
		OCP, Atlantik, CEM > BD > PR MTA > control	Runx2, ALP	7 days	*p* < 0.05
		OCP > CEM, Atlantik > BD, PR MTA, control	ALP	3 days	*p* < 0.05
		OCP > CEM, PR MTA > BD, control > Atlantik	DSPP	3 days	*p* < 0.05
		PR MTA > BD, CEM > OCP > Atlantik > control		7 days	*p* < 0.05
		BD > PR MTA > CEM > OCP, Atlantik, control	IL-Iα	3 days	*p* < 0.05
		PR MTA > CEM, BD, OCP, Atlantik, control		7 days	*p* < 0.05
		BD > CEM > PR MTA > OCP, Atlantik, control	IL-Iβ	3 days	*p* < 0.05
		PR MTA > BD > CEM >OCP, Atlantik, control		7 days	*p* < 0.05
		Atlantik > BD > CEM, PR MTA, OCP > control	IL6	3 days	*p* < 0.05
		CEM > BD, Atlantik > PR MTA, OCP > control		7 days	*p* < 0.05
		PR MTA > OCP, Atlantik > BD, CEM > control	TNFα	3 days	*p* < 0.05
		Atlantik > PR MTA, BD, OCP > CEM > control		7 days	*p* < 0.05
Miller et al. 2018 [34]	qRT-PCR	ES > Es FS	ALP, DSPP	21 days	*p* < 0.01
		BD > PR MTA	ALP	21 days	*p* < 0.05
			DSPP	21 days	*p* < 0.001
		PR MTA > ES, BD, ES FS	IBSP	21 days	*p* < 0.05
		PR MTA, BD, ES > control	IBSP	21 days	*p* < 0.05
Hajizadeh et al. 2018 [35]	qRT-PCR	CEMb > control	SP7, DSPP	2 weeks	*p* < 0.05
		PR MTA > control	ALP, SP7	2 weeks	*p* < 0.05
		Control > CEMb, PR MTA	ALP, SP7	3 weeks	*p* < 0.05
		Control > CEMb	DSPP, OSC	3 weeks	*p* < 0.05
Wongwatanasanti et al. 2018 [36]	qRT-PCR	BD > PR MTA, R MTA	DMP-1	14, 21 days	*p* < 0.05
		R MTA, BD > PR MTA	DSPP	14 days	*p* < 0.05
		BD, PR MTA > R MTA	DSPP, MEPE	21 days	*p* < 0.05
		PR MTA > BD	OCN	7 days	*p* < 0.05
		BD > PR MTA	MEPE	14 days	*p* < 0.05
Bi et al. 2018 [38]	qRT-PCR	iRoot FM > control, Ca(OH)_2_, TAP	ALP	10 days	*p* < 0.01
		iRoot FM > control	DMP-1	10 days	*p* < 0.005
		iRoot FM > Ca(OH)_2_, TAP		10 days	*p* < 0.01
	Western blot	iRoot FM > control, Ca(OH)_2_, TAP	ALP	10 days	*p* < 0.01
			DMP-1	10 days	*p* < 0.005
Peters et al. 2015 [39]	ELISA	PR MTA, BD > control	VEGF	3 days	*p* < 0.05
		Control > PR MTA, BD	ANGPT-1	3 days	*p* < 0.05
	qRT-PCR	PR MTA, BD > control	VEGFA, FGIF	3 days	*p* < 0.05
		Control > PR MTA > BD	ANGPT1, FGF2	3 days	*p* < 0.05
		BD > control, PR MTA	TGFβ1	3 days	*p* < 0.05
Yan et al. 2014 [41]	qRT-PCR	PR MTA > control	ALP, DSPP, RUNX2, OCN, IL-Iα. IL-Iβ, IL6	3, 7 days	*p* < 0.05

**Table 6:** Summary of the results of included studies showing significant differences between bioceramic materials or a bioceramic material and a control for hSCAP mineralization potential assays.

Author	Significant Results	Duration	Significance Level
Liu et al. 2019 [32]	iRoot FS > PR MTA	4 weeks	*p* > 0.05
	iRoot FS > control	4 weeks	*p* < 0.01
	PR MTA > control	4 weeks	*p* < 0.01
Saberi et al. 2019 [33]	PR MTA, BD, CEM, Atlantik, OCP > control	21 days	*p* < 0.05
Miller et al. 2018 [34]	PR MTA, BD, ES > control	21 days	*p* < 0.05
Wongwatanasanti et al. 2018 [36]	BD > PR MTA, R MTA, control	7, 14, 21 days	*p* < 0.05
Bi et al. 2018 [38]	iRoot FM > control, TAP	4 weeks	*p* < 0.005
	iRoot FM > Ca(OH)_2_	4 weeks	*p* < 0.01
Yan et al. 2014 [41]	PR MTA > control	14 days	*p* < 0.01
